# Prognostic role of microRNAs in human gastrointestinal cancer: A systematic review and meta-analysis

**DOI:** 10.18632/oncotarget.16679

**Published:** 2017-03-29

**Authors:** Qiang Zheng, Changyu Chen, Haiyang Guan, Weibiao Kang, Changjun Yu

**Affiliations:** ^1^ Department of Gastrointestinal Surgery, First Affiliated Hospital of Anhui Medical University, Hefei, China; ^2^ Department of General Surgery, First Affiliated Hospital of Anhui Traditional Medical University, Hefei, China; ^3^ Hefei National Laboratory for Physical Sciences at Microscale and School of Life Sciences, University of Science and Technology of China, Hefei, China

**Keywords:** microRNAs, prognosis, gastrointestinal cancer, target, meta-analysis

## Abstract

**Background:**

Gastrointestinal cancers (GICs) mainly including esophageal, gastric and colorectal cancer, are the most common cause of cancer-related death and lead into high mortality worldwide. We performed this systematic review and meta-analysis to elucidate relationship between multiple microRNAs (miRs) expression and survival of GIC patients.

**Methods:**

We searched a wide range of database. Fixed-effects and random-effects models were used to calculate the pooled hazard ratio values of overall survival and disease free survival. In addition, funnel plots were used to qualitatively analyze the publication bias and verified by Begg's test while it seems asymmetry.

**Results:**

60 studies involving a total of 6225 patients (1271 with esophageal cancer, 3467 with gastric cancer and 1517 with colorectal cancer) were included in our meta-analysis. The pooled hazard ratio values of overall survival related to different miRs expression in esophageal, gastric, colorectal and gastrointestinal cancer were 2.10 (1.78-2.49), 2.02 (1.83-2.23), 2.54 (2.14-3.02) and 2.15 (1.99-2.31), respectively. We have identified a total of 59 miRs including 23 significantly up-regulated expression miRs (miR-214, miR-17, miR-20a, miR-200c, miR-107, miR-27a, etc.) and 36 significantly down-regulated expression miRs (miR-433, let-7g, miR-125a-5p, miR-760, miR-206, miR-26a, miR-200b, miR-185, etc.) correlated with poor prognosis in GIC patients. Moreover, 35 of them revealed mechanisms.

**Conclusion:**

Overall, specific miRs are significantly associated with the prognosis of GIC patients and potentially eligible for the prediction of patients survival. It also provides a potential value for clinical decision-making development and may serve as a promising miR-based target therapy waiting for further elucidation.

## INTRODUCTION

Gastrointestinal cancers (GICs) mainly including esophageal cancer (EC), gastric cancer (GC) and colorectal cancer (CRC), are the most common cause of cancer-related death leading into high mortality worldwide, and it is still among the highest threatening risk of public health for past decades [1]. Actually, GIC patients at early stage could be cured successfully by receiving proper treatment (adjuvant chemotherapy or radiotherapy after radical resection) following approximately 90% five-year overall survival rate. However, five-year overall survival rate will decline to merely 15% when develop into advanced stage [2, 3]. Therefore, early diagnosis and prediction of individual prognosis play pivotal roles in the treatment and recovery of patients. However, there still lack of effective methods to evaluate the prognosis of GIC patients based on clinicopathology. Currently, increasing studies have reported that aberrant expression of specific microRNAs (miRs) as stable molecular biomarkers was associated with the prognosis of GIC patients and related to the targeted therapy, which provides potentially novel prevention strategies and advanced therapies [4–6].

Recently, near 8000 human miRs are registered in miRBase (http://www.mirbase.org/), and they regulate approximately 30% of all gene expression [7]. MiR is a short (20-24 nucleotides) class of non-coding RNA that can target 3′-untranslated regions (3′-UTRs) of mRNA and regulate its expression by degrading a mRNA or suppressing its translation [8, 9]. Additionally, one kind of miR can target several kinds of mRNAs at post-transcriptional level. For example, upregulated miR-377 expression promotes tumor proliferation by targeting P53, PTEN and TIMP1 [10]. Meanwhile, various miRs could target identical gene. Furthermore, miR plays a key role in the proliferation and progression of tumor cells, which not only mediates the cells growth, invision, migration and apoptosis but also induces resistance of anticancer drug [11]. For example, down-regulated miR-23b-3p induces chemo-resistence of gastric cancer cells [12]. In addition, many studies have reported that different miRs can be prognostic biomarkers in a wide range of human cancers (ovarian cancer, breast cancer, esophageal cancer, etc.) [13–17].

At present, accumulative evidences have demonstrated that abnormal expression of miRs as stable molecular biomarkers presented potential huge prognostic values in GIC patients [18–23]. However, these mono-centric, small sample size studies and various experimental protocols from different research departments limited the ability of evaluating relationship between multiple miRs expression and prognosis of GIC patients. The aim of this paper was to elucidate relationship between multiple miRs expression and prognosis of patients and investigate the possible utility of miRs as prognostic biomarkers in GIC patients. Moreover, further understanding of prognostic value of miRs could help for clinical decision-making and develop miR-based target therapeutic treatments.

## RESULTS

### Study identification and characteristics

**Figure 1:**
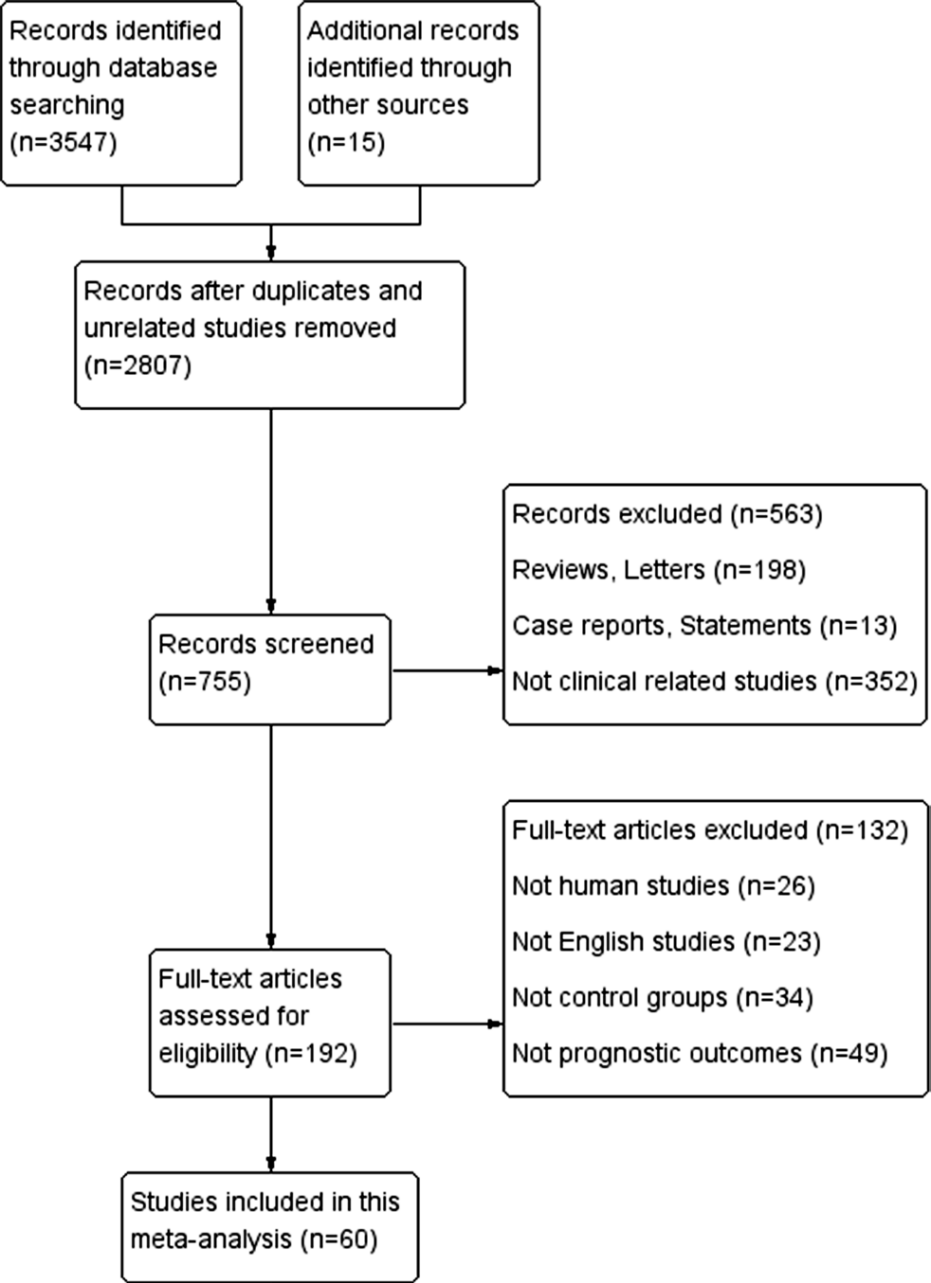
Study flow diagram

60 studies (12 EC, 35 GC and 13 CRC) involving a total of 6255 patients (1271 with EC, 3467 with GC and 1517 with CRC) were included in our meta-analysis based on selection criteria and specific steps were presented in Figure 1 [1, 2, 10–12, 16, 18–71]. More than half of included studies were from East Asian countries. Detection methods of miRs expression were mostly reverse transcription PCR (RT-PCR) or in sit hybridization (ISH) or microarray. Cut-off values of high or low miRs expression were mainly mean and median values. As for clinical endpoints, there were 46 studies [1, 2, 10–12, 16, 20, 22, 24–27, 29–33, 36–41, 43–59, 61–66] including overall survival (OS), 5 studies [67–71] including disease free survival (DFS) and another 9 studies [18, 19, 21, 23, 28, 34, 35, 42, 60] including both OS and DFS. We have identified a total of 59 miRs including 23 significantly up-regulated expression miRs (miR-214, miR-17, miR-20a, miR-200c, miR-107, miR-27a, miR-196b, miR-222, miR-106b, miR-500, miR-377, miR-25, miR-181a, miR-183, miR-1288, miR-106a-5p, miR-21, miR-30e, miR-142-3p, miR-1246, miR-508, miR-503, miR-942) and 36 significantly down-regulated expression miRs (miR-433, let-7g, miR-125a-5p, miR-760, miR-206, miR-26a, miR-200b, miR-185, miR-22, miR-217, miR-506, miR-133, miR-218, miR-137, miR-326, miR-486-5p, miR-29, miR-23b-3p, miR-194, miR-451, miR-34a, miR-106a, miR-143, miR-16, miR-139-3p, miR-361-5p, miR-365, miR-338-3p, miR-200c, miR-141, miR-150, miR-138, miR-134a, miR-195, miR-203, miR-375) correlated with poor prognosis in GIC patients (Table 1, Table 2). Moreover, 35 of them revealed mechanisms (Table 3).

**Table 1:** Characteristics of studies and different miRs expression related to OS in GIC patients

References	Year	MiRs	Nations	Number	OS		Cut-off	Detection	Sample	Follow
		(*n*= 54)		(*n*= 6125)	HR*	95% CI	value	methods	types	up
Schetter [24]	2008	miR-21↑	USA	CRC 71	2.70*	1.30-5.50	Third tertile	RT-PCR	tissue	<80
		miR-21↑	China	CRC 103	2.40*	1.40-4.10	Dichotomize	Microarray	tissue	<80
Mathé [25]	2009	miR-21↑	USA	EC 69	4.71*	1.74-12.79	Dichotomize	RT-PCR	tissue	<60
Toiyama [26]	2013	miR-21↑	Japan	CRC 168	4.12*	1.10-15.40	YI	RT-PCR	serum	<60
Oue [27]	2014	miR-21↑	Japan	CRC 156	1.80*	0.91-3.58	Third tertile	RT-PCR	tissue	<60
^a^Wang [16]	2015	miR-21↑	China	GC 50	1.89	1.17-3.07	mean	RT-PCR	tissue	<12
Hu [28]	2011	miR-30e↑	China	EC 158	1.80	1.26-2.57	median	ISH	tissue	1-256
Lin [29]	2012	miR-142-3p↑	China	EC 91	1.90*	1.10-3.31	median	RT-PCR	tissue	<70
Yokobori [30]	2012	miR-150↓	Japan	EC 108	**1.71**	0.88-3.33	median	RT-PCR	tissue	1-128
Gong [31]	2013	miR-138↓	China	EC 205	**1.76**	1.20-2.59	median	RT-PCR	tissue	<120
Takeshita [32]	2013	miR-1246↑	Japan	EC 101	4.03	1.28-12.73	median	RT-PCR	serum	<24
Akanuma [33]	2014	miR-134a↓	Japan	EC 84	**2.05**	1.02-4.11	median	RT-PCR	tissue	<120
Lin [34]	2014	miR-508↑	China	EC 207	**3.12**	2.06-4.75	median	RT-PCR	tissue	<60
Sun [35]	2014	miR-195↓	China	EC 98	5.96	1.26-11.93	median	RT-PCR	tissue	1-63
Ide [36]	2015	miR-503↑	Japan	EC 61	4.13*	1.47-11.33	median	RT-PCR	tissue	<80
Ge [37]	2015	miR-942↑	China	EC 158	**1.88**	1.19-2.96	median	RT-PCR	tissue	<80
Ueda [38]	2010	miR-214↑	Japan	GC 184	2.40	1.20-4.50	median	RT-PCR	tissue	5-102
		miR-433 ↓			2.10	1.10-3.90	median	RT-PCR	tissue	5-102
		let-7g↓			2.60	1.30-4.90	median	RT-PCR	tissue	5-102
Nishida [39]	2011	miR-125a-5p↓	Japan	GC 87	**1.87**	0.95-3.66	mean	RT-PCR	tissue	1-148
Ayerbes [1]	2011	miR-17↑	Spain	GC 38	2.62	1.55-4.49	mean	RT-PCR	BM	1-97
Wang [20]	2012	miR-17-5p↑	China	GC 65	1.79	1.11-2.87	median	RT-PCR	plasma	<34
		miR-20a↑			1.58*	1.10-2.25	median	RT-PCR	plasma	<36
Ayerbes [18]	2012	miR-200c↑	Spain	GC 52	2.24*	1.09-4.61	mean	RT-PCR	blood	6-53
Inoue [23]	2012	miR-107↑	Japan	GC 161	0.45*	0.22-0.85	mean	RT-PCR	tissue	6-72
Iwaya [40]	2013	miR-760↓	Japan	GC 82	1.67	1.03-3.11	median	RT-PCR	BM	<72
Yang [2]	2013	miR-206↓	China	GC 98	2.60	1.80-5.80	mean	RT-PCR	tissue	6-139
Deng [41]	2013	miR-26a↓	China	GC 126	**2.55**	1.57-4.16	2 fold	ISH	tissue	24-60
Tang [42]	2013	miR-200b↓	China	GC 36	**2.08**	1.28-3.37	2 fold	ISH	tissue	23-59
Tan [43]	2013	miR-185↓	China	GC 36	**2.33**	0.99-5.47	median	ISH	tissue	32-58
Huang [44]	2013	miR-27a↑	China	GC 82	1.75	1.02-3.01	NR	RT-PCR	serum	<20
Lim [45]	2013	miR-196b↑	China	GC 60	**1.87**	0.17-20.14	median	Microarray	tissue	35-76
Wang [46]	2013	miR-22↓	China	GC 98	2.20*	0.60-5.20	mean	RT-PCR	tissue	6-139
Fu [21]	2014	miR-222↑	China	GC 114	3.41*	1.84-6.16	median	RT-PCR	plasma	18-60
Yang [47]	2014	miR-106b↑	China	GC 120	**1.64**	1.02-2.61	median	Microarray	tissue	2-40
Cheng [48]	2014	miR-133↓	China	GC 180	**1.85**	0.60-5.70	mean	RT-PCR	tissue	38-60
Xin [49]	2014	miR-218↓	China	GC 68	3.16*	1.06-9.40	mean	RT-PCR	serum	<36
Chen [50]	2015	miR-217↓	China	GC 83	2.63	1.18-4.34	median	RT-PCR	tissue	<90
Zhang [51]	2015	miR-500↑	China	GC 323	2.23	1.66-3.23	median	RT-PCR	tissue	<60
Deng [52]	2015	miR-506↓	China	GC 63	**1.53**	0.53-4.39	median	RT-PCR	tissue	22-77
Gu [22]	2015	miR-137↓	China	GC 87	3.74	1.81-7.73	median	RT-PCR	tissue	<96
Li [53]	2015	miR-326↓	China	GC 136	1.51*	1.08-2.76	median	RT-PCR	tissue	8-93
Chen [54]	2015	miR-486-5p↓	China	GC 84	3.61*	1.99-6.54	median	ISH	tissue	1-75
^a^Wang [16]	2015	miR-29↓	China	GC 50	2.23	1.34-3.65	mean	RT-PCR	tissue	<12
WEN [10]	2015	miR-377↑	China	GC 102	2.14*	0.87-4.42	mean	RT-PCR	tissue	<60
Gong [55]	2015	miR-25↑	China	GC 40	**2.04**	0.80-5.10	mean	RT-PCR	tissue	36-61
An [12]	2015	miR-23b-3p↓	China	GC 140	**2.07**	1.14-3.76	NR	ISH	tissue	1-56
Chen [56]	2015	miR-194↓	China	GC 76	**3.23**	1.20-8.71	mean	RT-PCR	tissue	26-84
Su [57]	2015	miR-451↓	China	GC 107	**1.03**	0.52-2.02	mean	RT-PCR	tissue	19-74
Shi [58]	2015	miR-206↓	China	GC 220	6.82*	1.51-21.29	mean	RT-PCR	tissue	<60
Hui [59]	2015	miR-34a↓	China	GC 76	2.33*	1.10-4.93	median	RT-PCR	tissue	<60
Imaoka [19]	2015	miR-203↓	Japan	GC 130	4.51*	1.23-23.69	YI	RT-PCR	serum	1-78
Diaz [60]	2008	miR-106a↓	Spain	CRC 110	1.90*	0.93-3.80	median	RT-PCR	tissue	68-99
Nishimura [61]	2012	miR-181a↑	Japan	CRC 162	2.36	0.81-6.85	median	RT-PCR	tissue	36-60
Guo [11]	2013	miR-143↓	China	CRC 79	2.73	0.68-10.96	median	RT-PCR	tissue	41-122
Zhou [62]	2013	miR-183↑	China	CRC 94	2.75*	1.12-6.33	mean	Microarray	tissue	<70
Qian [63]	2013	miR-16↓	China	CRC 143	2.59	2.14-3.35	mean	RT-PCR	tissue	<120
Liu [64]	2014	miR-139-3p↓	China	CRC 63	2.79*	1.01-7.76	mean	RT-PCR	tissue	<80
Ma [65]	2014	miR-361-5p↓	China	CRC 60	**2.24**	0.48-10.50	mean	RT-PCR	tissue	3-60
Gopalan [66]	2014	miR-1288↑	Australia	CRC 122	**1.61**	0.14-19.23	2-fold	RT-PCR	tissue	10-68

^a^ One study involved both miR-29 and miR-21; * = adjusted HR;↑or↓ up-regulated or down-regulated with poor prognosis; GIC gastrointestinal cancer; EC esophageal cancer; GC gastric cancer; CRC colorectal cancer; OS overall survival; HR hazard ratio; NR not report; YI Youden index; RT-PCR reverse transcription PCR; ISH in sit hybridization; BM bone marrow; Calculated HR of OS was in bold.

**Table 2:** Characteristics of studies and different miRs expression related to DFS in GIC patients

References	Year	MiRNAs	Nations	Number	DFS		Cut-off	Detection	Sample	Follow
		(*n*=15)		(*n*=1373)	HR*	95% CI	value	methods	types	up
#Diaz [60]	2008	miR-106a↓	Spain	CRC 110	**2.80**	1.30-60	median	RT-PCR	tissue	68-99
Nguyen [67]	2010	miR-375↓	USA	EC 58	2.73*	1.17-6.39	median	RT-PCR	tissue	<80
#Hu [28]	2011	miR-30e↑	China	EC 158	1.67	1.17-2.38	median	RT-PCR	tissue	1-256
#Ayerbes [18]	2012	miR-200c↑	Spain	GC 52	2.27*	1.09-4.71	mean	RT-PCR	blood	6-53
#Inoue [23]	2012	miR-107↑	Japan	GC 161	0.14*	0.01-0.67	mean	RT-PCR	tissue	6-72
Nie [68]	2012	miR-365↓	China	CRC 76	**1.84**	0.80-4.22	mean	RT-PCR	tissue	1-38
#Tang [42]	2013	miR-200b↓	China	GC 36	**1.57**	0.97-2.53	2 fold	ISH	tissue	23-59
#Fu [21]	2014	miR-222↑	China	GC 114	3.38*	1.87-5.23	median	RT-PCR	plasma	18-60
#Lin [34]	2014	miR-508↑	China	EC 207	**3.92**	2.68-5.75	median	RT-PCR	tissue	<60
#Sun [35]	2014	miR-195↓	China	EC 98	5.59	1.13-11.16	median	RT-PCR	tissue	1-63
Sun [69]	2014	miR-338-3p↓	China	CRC 40	2.30	1.20-3.90	mean	RT-PCR	tissue	1-72
#Imaoka [19]	2015	miR-203↓	Japan	GC 130	**1.54**	0.46-5.13	YI	RT-PCR	serum	1-78
Zhou [70]	2015	miR-200c↓	China	GC 63	**1.38**	0.70-2.72	median	RT-PCR	tissue	28-33
		miR-141↓			**1.20**	0.58-2.46	median	RT-PCR	tissue	28-33
Yue [71]	2015	miR-106a-5p↑	China	CRC 70	2.21	1.46-4.11	median	RT-PCR	tissue	<80

# = Studies included both OS and DFS; * = adjusted HR;↑or↓ up-regulated or down-regulated with poor prognosis; GIC gastrointestinal cancer; EC esophageal cancer; GC gastric cancer; CRC colorectal cancer; OS overall survival; DFS disease free survival; HR hazard ratio; YI Youden index; RT-PCR reverse transcription PCR; ISH in sit hybridization; Calculated HR of DFS was in bold.

**Table 3:** miRs and target genes in gastrointestinal cancer

MiRs (n=35)	Poor prognosis	Role	Target genes	Function	Reference
miR-21↑	Up-regulation	oncogene	PTEN, TIMP1	growth/invasion/migration/apoptosis	[16, 24–27]
miR-107↑	Up-regulation	oncogene	DICER1	invasion/migration	[23]
miR-377↑	Up-regulation	oncogene	P53, PTEN, TIMP1	proliferation	[10]
miR-25↑	Up-regulation	oncogene	FBXW7	growth/invasion/migration	[55]
miR-106b↑	Up-regulation	oncogene	PTEN	invasion/migration	[47]
miR-500↑	Up-regulation	oncogene	NF-ĸB	proliferation/apoptosis	[51]
miR-181a↑	Up-regulation	oncogene	PTEN	proliferation	[61]
miR-183↑	Up-regulation	oncogene	PTEN	migration	[62]
miR-508↑	Up-regulation	oncogene	INPP5J	growth/invasion/migration	[34]
miR-942↑	Up-regulation	oncogene	sFRP4, GSK3β, TLE1	growth	[37]
miR-1288↑	Up-regulation	oncogene	FOXO1	proliferation	[66]
miR-137↓	Down-regulation	suppressor	AKT2	growth	[22]
miR-138↓	Down-regulation	suppressor	NF-kB	growth	[31]
miR-760↓	Down-regulation	suppressor	HIST1H3D	migration	[40]
miR-326↓	Down-regulation	suppressor	FSCN1	growth/migration	[53]
miR-125a-5p↓	Down-regulation	suppressor	ERBB2	growth	[39]
miR-134a↓	Down-regulation	suppressor	FSCN, MMP14	invasion/migration	[33]
miR-150↓	Down-regulation	suppressor	ZEB1	EMT	[30]
miR-217↓	Down-regulation	suppressor	EZH2	progression/metastasis	[48]
miR-506↓	Down-regulation	suppressor	Yap1	proliferation/invasion	[52]
miR-26a↓	Down-regulation	suppressor	FGF9	growth/metastasis	[41]
miR-200b↓	Down-regulation	suppressor	DNMT3A/3B, SP1	growth	[42]
miR-23b-3p↓	Down-regulation	suppressor	ATG12, HMGB2	chemoresistance	[12]
miR-133↓	Down-regulation	suppressor	CDC42–PAK	growth/migration/invasion	[48]
miR-185↓	Down-regulation	suppressor	DNMT1, CDC42	metastasis	[43]
miR-194↓	Down-regulation	suppressor	RBX1	proliferation/migration	[56]
miR-218↓	Down-regulation	suppressor	Robo1	growth/invasion/apoptosis	[49]
miR-200c/141↓	Down-regulation	suppressor	ZEB1/2	migration/ invasion	[70]
miR-143↓	Down-regulation	suppressor	TLR2	invasion/migration	[11]
miR-106a↓	Down-regulation	suppressor	EGFL7, E2F1	invasion/migration	[60]
miR-365↓	Down-regulation	suppressor	Cyclin D1, Bcl-2	apoptosis	[68]
miR-16↓	Down-regulation	suppressor	P53	growth	[64]
miR-338-3p↓	Down-regulation	suppressor	SMO	apoptosis	[69]
miR-203↓	Down-regulation	suppressor	E-cadherin	EMT/migration	[19]

### Meta-analysis findings

**Figure 2:**
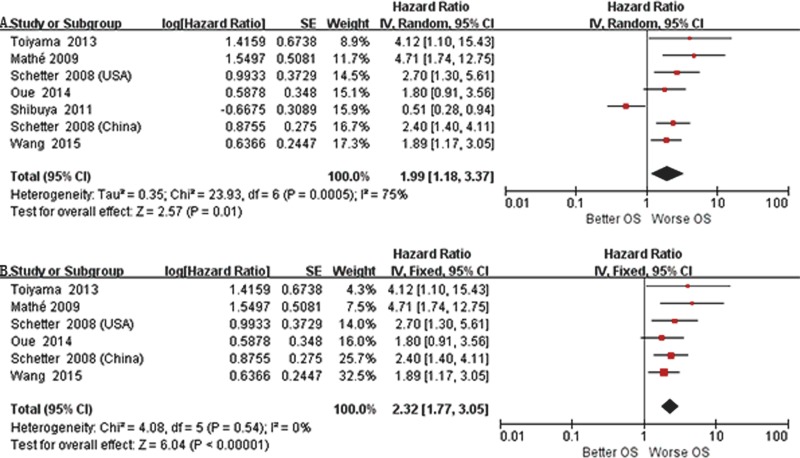
We performed forest plot to evaluate that the pooled hazard ratio value (95% CI) of overall survival related to expression level of miR-21 in gastrointestinal cancer patients **A**. Random-effects model, **B**. Fixed-effects model.

**Figure 3:**
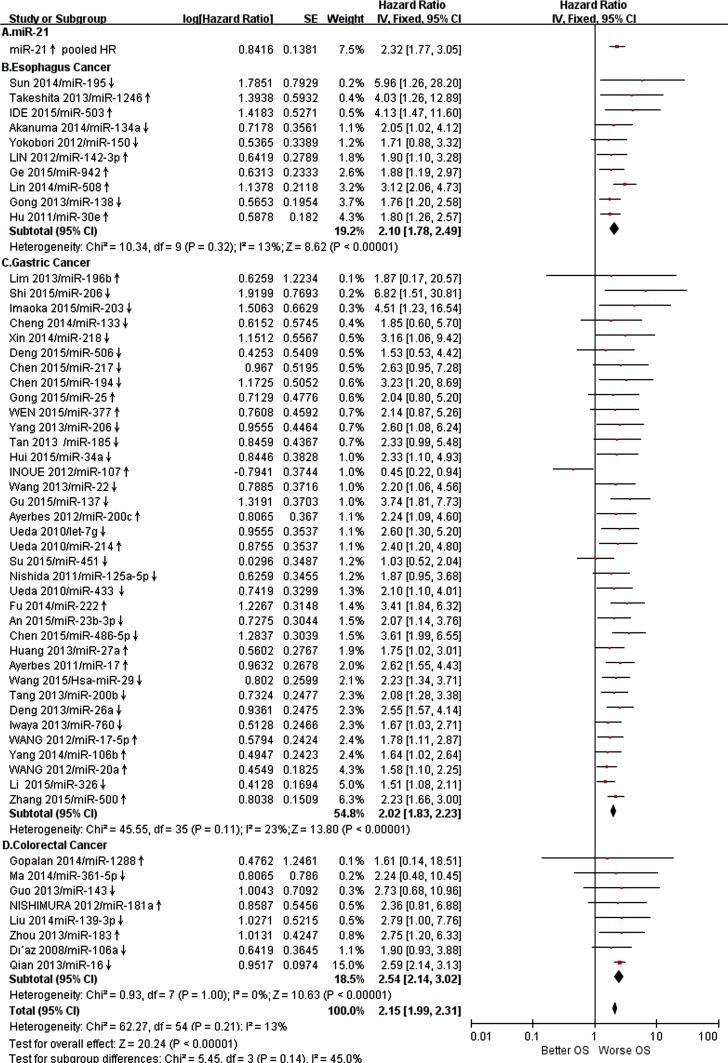
Forest plot of OS associated with expression level of different miRs in GIC patients was presented **A**. Pooled miR-21 expression in GIC, **B**. Specific miRs expression in EC, **C**. Specific miRs expression in GC, **D**. Specific miRs expression in CRC. OS overall survival; GIC gastrointestinal cancer; EC esophageal cancer; GC gastric cancer; CRC colorectal cancer.

**Figure 4:**
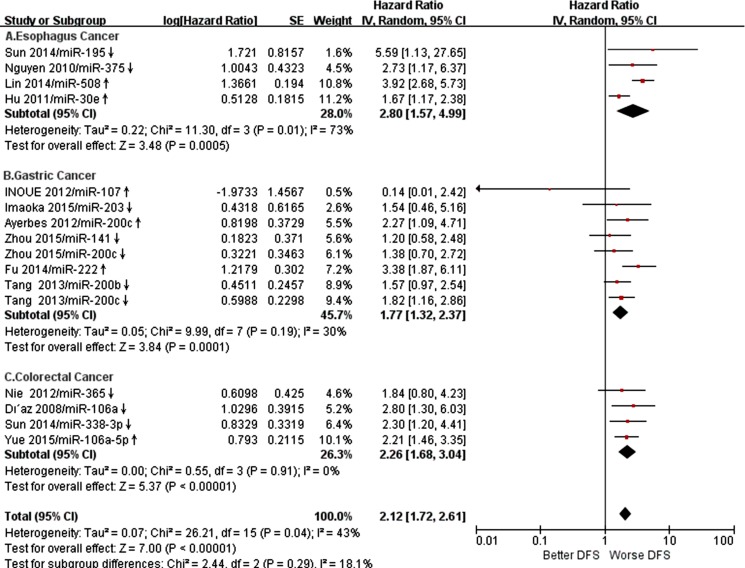
Forest plot of DFS associated with expression level of specific miRs in GIC patients was presented **A**. Specific miRs expression in EC, **B**. Specific miRs expression in GC, **C**. Specific miRs expression in CRC. DFS disease free survival; GIC gastrointestinal cancer; EC esophageal cancer; GC gastric cancer; CRC colorectal cancer.

**Figure 5:**
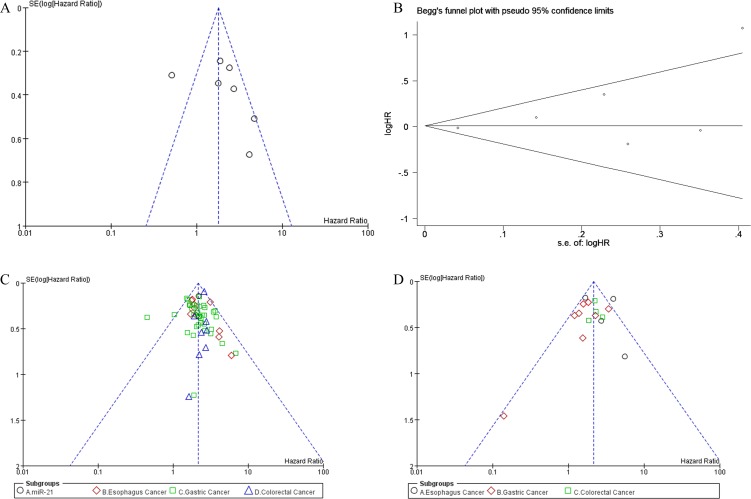
Funnel plots of included studies in this meta-analysis **A**. highly expressed miR-21 correlated with OS in GIC patients, **B**. highly expressed miR-21 correlated with OS in GIC patients was verified by Begg's test, **C**. Aberrantly expressed miRs correlated with OS in GIC patients, **D**. Aberrantly expressed miRs correlated with DFS in GIC patients. OS overall survival; DFS disease free survival; GIC gastrointestinal cancer.

We applied both random-effects and fixed-effects models to evaluate that the pooled hazard ratio (HR) value (95% CI) of OS was 2.32 (1.77-3.05) related to expression level of miR-21 in GIC patients with low heterogeneity (*P* =0.54, *I*2 =0%) and statistically significance (*P* <0.00001) after excluded one study [16, 24–27] (Figure 2). For all included studies, pooled HR values (95% CI) of OS related to different miRs expression in EC, GC, CRC and GIC patients were 2.10 (1.78-2.49), 2.02 (1.83-2.23), 2.54 (2.14-3.02) and 2.15 (1.99-2.31), respectively. And there was low heterogeneity (*P* =0.21, *I*2 =13%) and statistically significance (*P* <0.00001) in GIC (Figure 3). Additionally, pooled HR value (95% CI) of DFS related to different miRs expression in GIC patients was 2.12 (1.72-2.61) with low heterogeneity (*P* =0.04, *I*2 =43%) and statistically significance (*P* <0.00001) (Figure 4). Pooled HR value of OS related to circulatory miRs expression in GIC patients was 2.02 (1.63-2.49) (Supplementary Figure 1). Furthermore, miR-21 related meta-analysis was verified by Begg's test (*P*=0.260) (Figure 5).

## DISCUSSION

Gastrointestinal cancer is still a deadly threat in human health due to tumor metastasis and relapse inducing refractory advanced tumor stage and poor prognosis. Yan et al. [72] have demonstrated that there was 40%-65% recurrence rate due to distant metastases and regional relapse in GC patients. Recently, numerous studies focused on the miRs as prognostic molecular biomarkers in GIC patients for precise prediction. For example, Kang et al. [73] reported that miR-21 can be an independent predictor for tumor relapse in CRC patients, and Xu et al. [74] demonstrated that miR-21 as a promising biomarker can predict the lymph node metastases of tumor in GC patients.

The pooled HR value of OS correlated with different miRs expression in GIC patients was 2.14 (1.98-2.30), which implied specific miRs as independent risks inducing poor prognosis and could be considered as prognostic indicators for clinical decision-making. OS was defined as the time interval between GIC confirmed and end of follow up [75]. Moreover, elevated miR-21 expression promoted the tumor cell growth, invasion and migration, and inhibited its apoptosis by targeted PTEN and TIMP1, which was associated with low overall survival. Therefore, miR-21 as a stable molecular biomarker can be used to predict the prognosis of GIC patients. Additionally, miR-21 can also play a diagnostic role in GIC patients [76]. The pooled HR value of DFS associated with different miRs was 2.12 (1.72-2.61), which demonstrated different miRs leading to poor DFS and can be applied to monitor the therapeutic effects after receiving radical resection or chemotherapy. DFS was described as the time interval from GIC confirmed to relapse or end of follow up [68]. All included miRs were statistically significant associated with poor prognosis in GIC patients. Generally, the expression level of identical miR in GIC patients was consistent. For example, Yang et al. [2] reported that decreased miR-206 expression correlated with worse OS in GC patient and the finding was confirmed by Shi et al. [58]. While there were inversely results from different research institutions for identical miR associated prognosis of GIC patients. For instance, Ayerbes et al. [18] revealed that highly expressed miR-200c induced poor DFS in GC patients. Conversely, zhou et al. [70] demonstrated that low expression of miR-200c leaded to worse DFS in GC patients. Usually, evaluating prognosis of patients is inextricably bound to clinical decision-making. And researching signal pathways and target genes of miRs may promote the development of novel drug target therapies. Therefore, we summarized the miRs mechanism research associated with prognosis of GIC patients. We found 35 miRs associated with prognosis of GIC patients had explicit targets and some of them have established animal models but further study on clinical trials is required.

Based on this meta-analysis, we can preliminarily draw the clinical value of multiple miRs correlated with prognosis of GIC patients. (1) Aberrant expression of different miRs was associated with the survival of patients and miR-21 as a stable molecular biomarker can predict the individual prognosis through detecting its expression levels in GIC patients. (2) MiRs can offer more precise information for clinical decision-making comparing with the clinicopathological characteristics (such as tumor grade and size) of GIC patients. (3) Expression levels of specific miRs can be detected in tumor tissues or blood samples, which can be used to monitor the therapeutic effects of GIC patients after receiving chemotherapy treatment. (4) Abnormal miRs expression may provide a clinically valuable application for identifying patients with high risk at early stage avoiding advanced cancer progression. (5) It also provides a potential value for clinical decision-making development and may serve as a promising miR-based target therapy waiting for further elucidation.

However, several limitations deserved focused. First, both detection methods (RT-PCR, ISH and microarray) and cut-off values (mean, median, etc.) were applied to evaluate the different miRs expression that may be the source of heterogeneity due to different algorithms. Second, several sample types (tissue, blood, serum, plasma and bone marrow) were researched by all included studies can also induce the heterogeneity. Quantifiable miRs can be obtained from tissue samples because of its endogenous expression and mostly used to predict the patient survival after receiving resection treatment. Circulatory miRs as noninvasive biomarkers were more likely to predict the prognosis of GIC patients at unresectable stage and surveille the treatment effects of receiving chemotherapy for long term follow up study when compared with tissue samples. Third, clinicopathology characteristics (American Joint Committee on Cancer stage, AJCC stage) associated with prognosis of GIC patients could be the confounding factors inducing high heterogeneity. Therefore, we merely included studies that were focusing on the full sages (I-IV) rather than one certain stage GIC research. Fourth, we extracted HR and 95% CI values from Kaplan-Meier curve according to Tierney's methodology because there were 21 studies lack of survival data, which may cause potential heterogeneity [77]. Fifth, more than half included studies that did not report the adjusted HR values were prone to high heterogeneity. As for publication bias, failure to publish negative results of articles leading to overestimate the pooled effect value, which have reached a consensus. Besides, language bias was existed because only English publications were enrolled in this study. Thus, we systematically searched a wide range of database and found there was no publication bias in all analysis except miR-21 related meta-analysis. After excluding one study in miR-21 related meta-analysis for sensitivity analysis, the pooled effect value did not substantially change implying high stability.

## CONCLUSIONS

Overall, specific miRs are significantly associated with the prognosis of GIC patients and potentially eligible for the prediction of patients survival. It also provides a potential value for clinical decision-making development and may serve as a promising miR-based target therapy waiting for further elucidation.

## MATERIALS AND METHODS

### Search strategy

We searched a wide range of database (PubMed, Web of Science and EMBASE) for published English articles, and additional records identified through other sources such as contacting authors and searching unpublished studies up to August 1, 2016. Search terms were consisted of “microRNA”, “miRNA”, “miR”, “cancer”, “tumor”, “malignant”, ”metastasis”, “carcinoma”, “gastrointestine”, “gastroenteric”, “esophagus”, “esophageal”, “gastric”, “stomach”, “colon”, “rectum”, “colonrectum”, “incidence”, “mortality”, “follow up studies”, “prognosis”, “prediction”, “survival”, “hazard ratio”, and combined with AND/OR.

### Selection criteria

Two reviewers read the studies intensively and evaluated the eligibility of studies independently based on selection criteria involving inclusion criteria: (1) Patients were diagnosed with gastrointestinal cancer by histopathology; (2) MiRs as prognostic markers were used to predict the prognosis for full stage (I-IV) patients. (3) Control group (healthy people or patients without GIC) was contained; (4) The effective outcomes were OS, DFS, HR and 95% CI; (5) Observational studies that we can extract the survival data from the articles or Kaplan-Meier survival curve were included; and exclusion criteria: (1) Non-English and non-human subject studies were excluded; (2) Studies were letters, reviews and reports lack of survival data; (3) Studies focused on genetic alterations about the polymorphisms or modification of miRs. We would get to consensus finally through discussion when disagreements came out.

### Data extraction and quality assessment

We collected specific information (the first author, year of publication, nation, number of patients, OS/DFS HR and 95% CI, cut-off value, detection method, sample type and follow up) from each included study. The quality of included studies was assessed according to the checklist of meta-analysis of observational studies in epidemiology (MOOSE) [78]:

Explicit definition of study population exposure.

Explicit definition of measurement of miRs expression such as qRT-PCR, ISH and microarray.

Explicit definition of outcomes (OS and DFS).

Explicit definition of cut-off value and follow-up.

Explicit definition of study design.

### Statistical analysis

Analysis was implemented by Review Manager 5.3 (The Nordic Cochrane Centre, The Cochrane Collaboration, London, UK) and Stata 12.0 (Stata Corporation, College Station, Texas, USA) software. We applied both fixed-effects and random-effects models to evaluate the pooled value of HR by calculating Cochran Q test and *I*2 Index values. If *P* >0.10 and *I*2 <50% implied that low heterogeneity of pooled HR value is statistically significant difference, fixed-effects model should be used finally. Otherwise, random-effects model would be performed. In addition, forest plots of pooled HR values were presented. Funnel plots were used to qualitatively analyze the publication bias and verified by Begg's test while it seems asymmetry. Moreover, we also conducted sensitivity analysis for this meta-analysis.

## SUPPLEMENTARY MATERIALS FIGURE


