# Supplementary material for: Prognostic role of microRNAs in human gastrointestinal cancer: A systematic review and meta-analysis
Source: Oncotarget. 2017 Mar 29;8(28):46611–23. doi: 10.18632/oncotarget.16679 (PMC5542297; doi:10.18632/oncotarget.16679)
Supplement: Supplementary file 1 [file oncotarget-08-46611-s001.pdf]

# Prognostic role of microRNAs in human gastrointestinal cancer: A systematic review and meta-analysis

## Supplementary Materials

| Study or Subgroup | log[Hazard Ratio] | SE | Weight | Hazard Ratio IV, Fixed, 95% CI |
|---|---|---|---|---|
| WANG 2012/miR-17-5p ↑ | 1.5063 | 0.6629 | 2.6% | 4.51 [1.23, 16.54] |
| WANG 2012/miR-20a ↑ | 1.3938 | 0.5932 | 3.3% | 4.03 [1.26, 12.89] |
| Ayerbes 2012/miR-200c ↑ | 1.1512 | 0.5567 | 3.8% | 3.16 [1.06, 9.42] |
| Imaoka 2015/miR-203 ↓ | 0.8065 | 0.367 | 8.6% | 2.24 [1.09, 4.60] |
| Xin 2014/miR-218 ↓ | 1.2267 | 0.3148 | 11.7% | 3.41 [1.84, 6.32] |
| Fu 2014/miR-222 ↑ | 0.5602 | 0.2767 | 15.2% | 1.75 [1.02, 3.01] |
| Huang 2013/miR-27a ↑ | 0.5794 | 0.2424 | 19.8% | 1.78 [1.11, 2.87] |
| Takeshita 2013/miR-1246 ↑ | 0.4549 | 0.1825 | 34.9% | 1.58 [1.10, 2.25] |
| **Total (95% CI)** | | | **100.0%** | **2.02 [1.63, 2.49]** |

Heterogeneity: $Chi^2 = 8.69$, df = 7 (P = 0.28); $I^2 = 19\%$
Test for overall effect: Z = 6.50 (P < 0.00001)

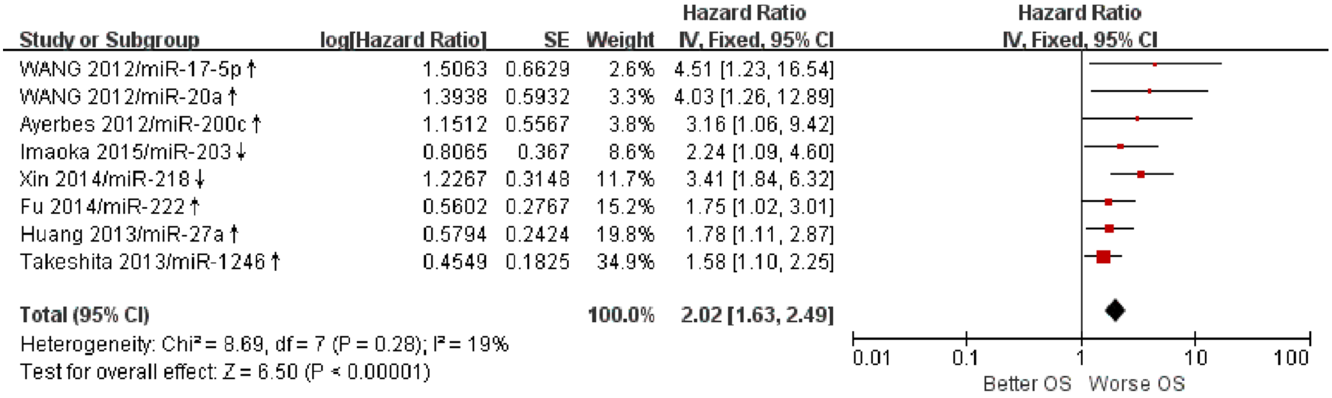


**Supplementary Figure S1: We performed forest plot to evaluate that the pooled hazard ratio value (95% CI) of overall survival related to expression level of circulatory miRs in gastrointestinal cancer patients.**
